# A study to establish reasonable action limits for patient‐specific quality assurance in intensity‐modulated radiation therapy

**DOI:** 10.1120/jacmp.v8i2.2374

**Published:** 2007-03-07

**Authors:** Stefan Both, Ionut M. Alecu, Andrada R. Stan, Marius Alecu, Andrei Ciura, Jeremy M. Hansen, Rodica Alecu

**Affiliations:** ^1^ Department of Radiation Oncology, Medical Physics Division University of Pennsylvania Philadelphia Pennsylvania; ^2^ Department of Chemistry, Physical Chemistry Division University of North Texas Denton Texas; ^3^ Department of Medical Physics AROS Colleyville Texas

**Keywords:** IMRT QA, fluence maps, acceptance level, percent of passing points, percent dose error

## Abstract

An effective patient quality assurance (QA) program for intensity‐modulated radiation therapy (IMRT) requires accurate and realistic plan acceptance criteria—that is, action limits. Based on dose measurements performed with a commercially available two‐dimensional (2D) diode array, we analyzed 747 fluence maps resulting from a routine patient QA program for IMRT plans. The fluence maps were calculated by three different commercially available (ADAC, CMS, Eclipse) treatment planning systems (TPSs) and were delivered using 6‐MV X‐ray beams produced by linear accelerators. To establish reasonably achievable and clinically acceptable limits for the dose deviations, the agreement between the measured and calculated fluence maps was evaluated in terms of percent dose error (PDE) for a few points and percent of passing points (PPP) for the isodose distribution. The analysis was conducted for each TPS used in the study (365 ADAC, 162 CMS, 220 Eclipse), for multiple treatment sites (prostate, pelvis, head and neck, spine, rectum, anus, lung, brain), at the normalization point for 3% percentage difference (%Diff) and 3‐mm distance to agreement (DTA) criteria. We investigated the treatment‐site dependency of PPP and PDE. The results show that, at 3% and 3‐mm criteria, a 95% PPP and 3% PDE can be achieved for prostate treatments and a 90% PPP and 5% PDE are attainable for any treatment site.

PACS Numbers: 87.53Dq, 87.53Tf, 87.53Xd, 87.56Fc

## I. INTRODUCTION

Extensive quality assurance (QA) for intensity‐modulated radiation therapy (IMRT) is essential for quality patient care in radiation therapy. Phantom measurements are routinely used for absolute and relative dose evaluations for patient IMRT QA.^(1)^ To ensure that IMRT plans are accurately delivered to the patients, phantoms containing film and ion chambers have traditionally been employed to prove that the measured and calculated doses are in agreement.^(^ ^2^ ^,^ ^3^ ^)^

Dong et al.^(3)^ extensively analyzed IMRT QA based on ion chamber measurements and found that accuracy in QA of up to ±7% and spatial accuracy of ±5 mm can be achieved. For some sites, those criteria can be lowered.

Diode arrays have also been used for IMRT QA. These devices are easy to use and provide QA results while measurements are being performed. Published studies^(^ ^4^ ^–^ ^6^ ^)^ provide evidence that diode arrays are an accurate and reliable tool for verification of IMRT treatment. The patient IMRT QA program described in this study is based on a commercial two‐dimensional (2D) N‐type semiconductor diode array that eliminates some of the steps involved in the combination of film and ion chamber dosimetry.

Validation of the fluence maps calculated by the treatment planning system (TPS) is a two‐step process:

1. The isodose distribution accuracy—that is, agreement between the measured and calculated relative doses—is evaluated by analyzing the values for percent of passing points (PPP), for percent difference (%Diff), and for distance to agreement (DTA).
  The PPP are the points that meet the criteria imposed by the %Diff and DTA constraints, as described by Nelms.^(^ ^7^ ^,^ ^8^ ^)^ The %Diff represents the allowed difference between measured and plan points with the same coordinates.^(8)^ The DTA is a radius specified in millimeters around the measured points, and it tests the plan dose points to determine if any lower and higher points are found within a certain radius around the measured point.^(8)^ The DTA is used when the difference between measured and plan points of the same coordinates exceed the set %Diff value. The threshold value represents the percent isodose line above which all plan results are included in the DTA analysis.^(8)^
2. Absolute doses delivered are evaluated. The discrepancy between the calculated and measured absolute dose in a point is quantified by the percent dose error (PDE) for each analyzed field.

It is widely accepted that the diode array provides evidence that the IMRT process is functioning within acceptable limits—that is, that the discrepancy between the delivered and prescribed dose is less than 5%.^(9)^ Our patient QA program is based solely on 2D diode array measurements for individual fields, with the beam perpendicular to the device for doses within the linear range of the device (<300 cGy^(^ ^4^ ^,^ ^6^ ^)^). We set the comparison criteria between a fluence map that was optimally calculated by a TPS and measured by the 2D diode array to ±3% for %Diff and ±3 mm for DTA at a 10% threshold. Here, we present the results of a preliminary study of 747 optimal fluence maps. The intent of the study was to establish the achievable level of dose agreement for a dose deviation that is clinically acceptable in an accurate, optimal patient treatment. We validated each individual field from the treatment plan rather than from the usual composite plan. This process is less time‐consuming and generally more stringent than is film composite analysis, in which small errors may be missed.^(5)^

## II. METHODS AND MATERIALS

The 6‐MV‐energy X‐ray beams from a linear accelerator [LINAC (Varian 21EX 120MLC: Varian Medical Systems, Palo Alto, CA)] were employed to deliver the optimal fluence maps calculated by three different TPSs—Varian Eclipse, CMS XiO (CMS, St. Louis, MO), and Phillips ADAC (Philips Medical Systems, Milpitas, CA)—for 747 fields across multiple treatment sites. A three‐dimensional (3D) water scanning system (Wellhofer Dosimetrie, Schwarzenbruck, Germany) with a cylindrical ion chamber (Model IC‐15: Wellhofer Dosimetrie, Schwarzenbruck, Germany) of 0.125 cm^3^ volume was used to collect the beam model data in accordance with published data.^(^ ^10^ ^,^ ^11^ ^)^ The dose verification for absolute and relative modes was performed using the 2D diode array (MapCheck Model 1175: Sun Nuclear, Melbourne, FL), which consists of 445 N‐type diodes in a variable spacing arrangement across a 22×22‐cm field. The 10×10‐cm center portion of the octagonal grid contains 221 diodes with 7‐mm spacing, and the outer area surrounding the central grid contains 224 diodes with 14‐mm spacing. The high spatial resolution of the N‐type improved silicone diodes (0.8×0.8 mm) makes them suitable for analysis of high dose gradient regions.^(8)^ Dose linearity was found to be up to 300 cGy.^(6)^

### A. MapCheck calibration

Calibration of the diode array was performed using a 6‐MV X‐ray LINAC beam calibrated at a 100‐cm source‐to‐surface distance (SSD), based on the TG‐51 protocol^(11)^ such that the calibration factor was ${\text{CF}}_{6X}=1\;\text{cGy}/\text{monitor}$ unit (MU).

The calibration of the 2D diode array was performed at a water equivalent depth of 4.5 cm (4.5 g/cm) that included a diode built‐in depth of 1.35 cm and an additional 2.4 cm of polystyrene.

#### A.1 Relative dose calibration

The relative dose calibration was performed using a 6‐MV X‐ray beam, delivering 100 MUs for a 26×26‐cm field size from a gantry angle of 0 degrees and 100‐cm SSD to the top of the device. The field size and SSD could be changed, provided that the ratio between the area of the calibration field and the diode array remained at about 1.1 (per scatter correction). The calibration method was adopted as described by the manufacturer,^(8)^ using a wide‐field method, and it consisted of multiple rotations and shifts of the device so as to determine the relative sensitivity between the array's detectors. These differences represent individual calibration factors, which are stored in a computer as relative calibration files for the array. Subsequently, the calibration files are applied to the array detector during relative measurements.

#### A.2 Absolute dose calibration

The setup for absolute dose calibration used a 10×10‐cm field size, a gantry angle of 0 degrees at 100.75‐cm SSD to the top of the added polystyrene plates. This setup is equivalent in water to a 100‐cm SSD and a detector depth of 4.50 cm. Using a 6‐MV X‐ray beam, 200 MUs were delivered. The absolute dose given to the diode at the central beam axis was calculated using the PDD value at 4.5‐cm water equivalent depth, multiplied by ${\text{CF}}_{6X}$. The determined dose calibration factor was stored in the computer as a reference calibration file for the beam used. The appropriate absolute calibration file was used during absolute dose determination.

### B. In‐phantom calculations and measurements

The comparison between each TPS‐calculated patient fluence map and the corresponding fluence map measured by the 2D diode array was performed based on the water equivalent concept. All fluence maps were calculated for a 95.5‐cm SSD, with the beam perpendicular to the phantom at a depth of 4.5 cm. To correct for the difference in the densities (device buildup and polystyrene versus homogeneous water phantom) and for beam divergence, the measurement setup was 96.25 cm at the top of the added polystyrene plates.

#### B.1 In‐phantom calculations

For calculation purposes, we created a homogeneous water density phantom for the CMS XIO and Varian Eclipse TPSs; the planar‐dose feature was used for the ADAC TPS. All IMRT QA plans were performed using an isocentric setup. For patient plan validation, the patient IMRT plan was transposed onto the phantom within the TPS. Each field was reset to deliver the dose with the beam perpendicular to the phantom from a gantry angle of 0 degrees, for a 95.5‐cm SSD. The weight of each beam was recalculated to deliver dose at the depth of 4.5 cm of water, for the same number of MUs as found in the original patient plan. The absolute doses table in a plane perpendicular to the central beam axis situated at the depth of 4.5 cm of water was generated and saved as an ASCII file. A hard copy of the phantom plan was generated for further evaluation.

#### B.2 In‐phantom measurements

The MapCheck was set up in the treatment room. A setup equivalent to the TPS setup was achieved by adding polystyrene to a thickness of 2.4 cm (total equivalent of 4.5 cm water) and by setting a 96.25‐cm SSD to the top of the added polystyrene plates. Each beam was delivered to the MapCheck using the same number of MUs and the same multileaf collimator file as in the original plan. All measurements were saved under the patient file for analysis purposes.

#### B.3 Validation of the QA procedure

A 10×10‐cm open field was used to validate the QA procedure. Once the phantom had been created in the TPS and the MapCheck calibration files had been saved, the agreement between the calculated and the measured optimal fluence maps was verified. For this purpose, a phantom plan—6‐MV X‐ray beam perpendicular to the homogeneous phantom, 10×10‐cm open field, 95.5‐cm SSD, and 4.5‐cm depth for a 100 cGy prescription to that depth—was created in the TPS. The plan was then transferred to the MapCheck and accelerator, geometry was reproduced with the SSD corrected for the differences in density, and dose was delivered as prescribed in the TPS.

#### B.4 Fluence map evaluation

The TPS dose plan file was imported into MapCheck. The measured and calculated fluence maps were both renormalized to the same point and then analyzed and compared at the relative dose distribution and absolute dose levels. The normalization point was set in the high dose, low dose gradient region. The measured and calculated fluence values were saved in a matrix file and compared. The absolute dose $\left(D_{\text{abs}}\right)$ was calculated as the product of the particular detector reading $\left(D_{i}\right)$ and the corresponding absolute calibration file (CF):

(1) $$D_{\text{abs}}=D_{i}\times \text{CF}.$$

We chose the criteria ±3% (%Diff) and 3 mm (DTA), with a threshold of 10%, as our clinical tolerance for each optimal fluence map calculated.

The measured doses were displayed in the first window, and the TPS calculated doses were displayed in the second window. A fluence map showing the location of the overexposed (red) and underexposed (blue) points was displayed in a third window. This latter window also showed the percentage of the measured points that satisfied the chosen %Diff and DTA criteria over the selected passing points for the 10% threshold. A forth window showing the measured and TPS‐calculated points for a particular dose profile was also generated. The PDE values at the central beam axis, normalization point, and a selected point were used to compute the PDE statistic. The results were printed for each beam and entered into the database for analysis.

## III. RESULTS AND DISCUSSIONS

### A. Validation of the QA procedure

For every TPS and LINAC used in this work, the results were 100% PPP for ±3% (%Diff) and 3‐mm (DTA) criteria for the relative dose distribution and 0.1%−0.5% PDE for the absolute doses, demonstrating that the whole QA chain was correctly set up.

### B. Fluence map evaluations

We conducted a Student *t*‐test statistical analysis^(12)^ of PPP and PDE values between head‐and‐neck and prostate and other localization cases (Table 1). The averaged PPP for prostate and other cases (99.3±1.41) was statistically significantly higher than the PPP for head‐and‐neck cases (96.22±2.89, p=0.00). The averaged PDE for prostate and other localizations cases (0.419±0.420) was statistically significantly lower than the PDE for head‐and‐neck cases (1.41±1.1, p=0.00). Variations in the PPP and PDE parameters were significantly less for prostate and other cases than for head‐and‐neck cases (p=0.00). These differences may be attributed to variation in target complexity and in shape and proximity of critical structures; sharper dose gradients must be planned and delivered in head‐and‐neck IMRT than in prostate IMRT. Our results suggest that a different criterion should be used for PPP and PDE parameters in prostate and other localizations than in head‐and‐neck localizations (Table 1).

**Table 1:** Statistical comparisons of the percent of passing points (PPP) and percent dose error (PDE) values between head‐and‐neck and prostate and other localizations

	Localization (mean±SD)		*t* Value	*p* Value
	Head‐and‐neck	Prostate and other		
PPP	96.22±2.89	99.30±1.41	18.43	0.00 ^a^
PDE	1.41±1.10	0.419±0.420	16.00	0.00 ^a^

a *p* Value<0.01 vs. 0.05.

SD=standard deviation.

**Figure 1:**
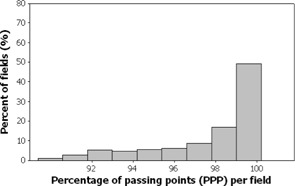
Overall results as a frequency distribution (total fields=747—that is, fluence maps for all localizations) of the percent of passing points measurements.

**Figure 2:**
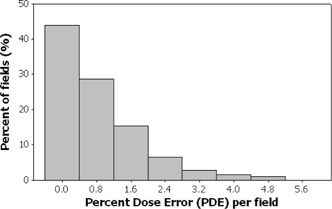
Histogram showing overall results as a frequency distribution (total fields=747—that is, fluence maps for all localizations) of the percent dose error measurements.

**Figure 3:**
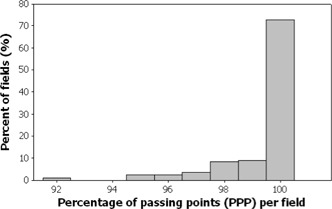
Selected results as a frequency distribution (total fields=389—that is, fluence maps for prostate and other localizations) of the percent of passing points measurements.

**Figure 4:**
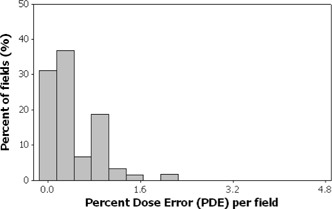
Histogram showing selected results as a frequency distribution (total fields=389—that is, fluence maps for prostate and other localizations) of the percent dose error measurements.

**Figure 5:**
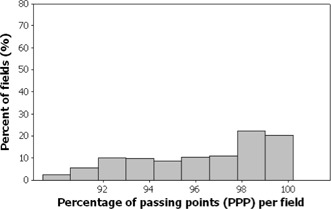
Selected results as a frequency distribution (total fields=358—that is, fluence maps for head and neck) of the percent of passing points measurements.

**Figure 6:**
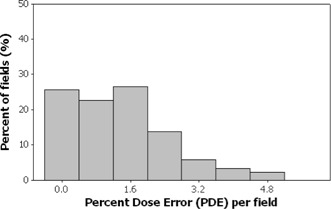
Histogram showing selected results as a frequency distribution (total fields=358—that is, fluence maps for head and neck) of the percent dose error measurements.

Figs. 1–6 present the PPP and PDE for the fluence maps measured and calculated by each TPS for the 3%, 3‐mm, and 10% criteria as histograms for all cases, for prostate and other cases, and for head‐and‐neck cases.

Figs. 1 and 2 illustrate the overall PPP and the PDE for 747 fluence maps calculated using the study criteria by the TPSs across all localizations. The percentage of fields with a PPP greater than 95% was 81.4% and with a PPP greater than 90% was 100% (Fig. 1). The percentage of fields with a PDE less than 3% was 95.9% and a PDE less than 5% was 100% (Fig. 2).

Figs. 3 and 4 illustrate the PPP and the PDE for 389 fluence maps calculated for prostate and other localizations excluding head and neck. The percentage of fields with a PPP of at least 95% was 96.4%; all are prostate cases. All fields had a PPP greater than 90% (Fig. 3). All fields had a PDE less than 3% (Fig. 4). Only 3.6% of the fields had a PPP value below 95%, and all fields met the PDE value of 3%.

Figs. 5 and 6 illustrate a total of 358 fluence maps calculated for head‐and‐neck localizations; all fall within the %Diff, DTA, and threshold study criteria. The percentage of fields with a PPP greater than 95% was 63.4%, and greater than 90% was 100% (Fig. 5). The percentage of fields with a PDE less than 3% was 94.1%, and less than 5% was 100% (Fig. 6). All fields passed with a PPP greater than 90% and a PDE less than 5%.

During the analysis, we investigated fields with a PDE greater 5% for the 3% and 3‐mm criteria. The differences between the planned and measured doses can be attributed to three different sources of error: dosimeter, delivery system and dose calculation system. However, if the dosimeter is properly chosen, commissioned, and maintained, errors related to calculation, delivery, or a combination of the two can be detected within the 5% accuracy goal of radiation therapy. The most common source of error was mistakes in setup (SSD, depth, orientation to gantry) or software (improper calibration file), and so were not reported. The PPP score depended on choice of normalization point. Profile analysis was a good indicator for choice of normalization point (high/low dose gradient) and permitted identification of the location of dose points not passing the chosen criteria (penumbra/plateau). Dose points situated in the penumbra region were found to be responsible for low PPP.

## IV. CONCLUSION

To summarize, with ±3% (%Diff), 3‐mm (DTA), and 10% (threshold) criteria as the minimum achievable, we found that 95% PPP and 3% PDE can be achieved in prostate IMRT. For other sites, 90% PPP and 5% PDE are realistic goals for the same %Diff, DTA, and threshold criteria. The achievable results found in the present study can therefore be considered to be criteria in the future, despite not knowing all sources of error.

## References

[1] Ezzell GA , Galvin JM , Low D , et al. Guidance document on delivery, treatment planning, and clinical implementation of IMRT: report of the IMRT Subcommittee of the AAPM Radiation Therapy Committee. Med Phys. 2003; 30 (8): 2089–2115. 12945975 10.1118/1.1591194

[2] Charland MP , Chetty IJ , Yokoyama S , Fraass BA . Dosimetric comparison of extended dose range film with ionization measurements in water and lung equivalent heterogeneous media exposed to megavoltage photons. JACMP. 2003; 4 (1): 25–39. 12540816 10.1120/jacmp.v4i1.2539 PMC5724437

[3] Dong L , Antolak J , Salehpour M , et al. Patient specific point dose measurement for IMRT monitor unit verification. Int J Radiat Oncol Biol Phys. 2003; 56 (3): 867–877. 12788197 10.1016/s0360-3016(03)00197-4

[4] Li JG , Dempsey JF , Ding L , Liu C , Palta JR . Validation of dynamic MLC‐controller log files using a two‐dimensional diode array. Med Phys. 2003; 30 (5): 799–805. 12772987 10.1118/1.1567951

[5] Jursinic PA , Nelms BE . A 2‐D diode array analysis software for verification of intensity modulated radiation therapy delivery. Med Phys. 2003; 30 (5): 870–879. 12772995 10.1118/1.1567831

[6] Letourneau D , Gulam M , Yan D , Oldham M , Wong JW . Evaluation of a 2D diode array for IMRT quality assurance. Radiother Oncol. 2004; 70 (2): 199–206. 15028408 10.1016/j.radonc.2003.10.014

[7] Nelms B , Simon W , Jursinic P . Verification of IMRT delivery using a 2‐D diode array and analysis software [Abstract]. Med Phys. 2002; 29 (6): 1364. 10.1118/1.1567831 12772995

[8] Sun Nuclear Corporation . MapCheck Model 1175 Manual. Melbourne (FL): Sun Nuclear Corporation; 2004.

[9] Kutcher GJ , Coia L , Gillin M , et al. Comprehensive QA for radiation oncology: report of AAPM Radiation Therapy Committee Task Group 40. Med Phys. 1994; 21 (4);581–618. 8058027 10.1118/1.597316

[10] Leybovich LB , Sethi A , Dogan N . Comparison of ionization chambers of various volumes for IMRT absolute dose verification. Med Phys. 2003; 30 (2): 119–132. 12607828 10.1118/1.1536161

[11] Almond PR , Biggs PJ , Coursey BM , et al. AAPM's TG‐51 protocol for clinical reference dosimetry of high‐energy photon and electron beams. Med Phys. 1999; 26 (9);1847–1870. 10505874 10.1118/1.598691

[12] Devore JL . Probability and Statistics for Engineering and the Sciences. 4th edition Belmont (CA): Duxbury Press; 1995.

